# Systematic pan-cancer analysis on the expression and role of regulator of chromatin condensation 1/small nucleolar RNA host gene 3/small nucleolar RNA host gene 12

**DOI:** 10.3389/fmolb.2022.946507

**Published:** 2022-09-06

**Authors:** Kai Hu, Huomei Yu, Shiyan Liu, Deyu Liao, Yan Zhang

**Affiliations:** Key Laboratory of Diagnostic Medicine of the Chinese Ministry of Education, School of Clinical Diagnostic and Laboratory Medicine, Chongqing Medical University, Chongqing, Yuzhong, China

**Keywords:** RCC1, SNHG3, SNHG12, pan-cancer, cell cycle

## Abstract

Regulator of chromatin condensation 1 (RCC1) is the major guanine nucleotide exchange factor of RAN GTPase, which plays a key role in various biological processes such as cell cycle and DNA damage repair. Small nucleolar RNA host gene 3 (SNHG3) and small nucleolar RNA host gene12 are long-stranded non-coding RNAs (lncRNAs) and are located on chromatin very close to the sequence of Regulator of chromatin condensation 1. Many studies have shown that they are aberrantly expressed in tumor tissues and can affect the proliferation and viability of cancer cells. Although the effects of Regulator of chromatin condensation 1/small nucleolar RNA host gene 3/small nucleolar RNA host gene12 on cellular activity have been reported, respectively, their overall analysis on the pan-cancer level has not been performed. Here, we performed a comprehensive analysis of Regulator of chromatin condensation 1/small nucleolar RNA host gene 3/small nucleolar RNA host gene12 in 33 cancers through the Cancer Genome Atlas and Gene Expression Database. The results showed that Regulator of chromatin condensation 1/small nucleolar RNA host gene 3/small nucleolar RNA host gene12 were highly expressed in a variety of tumor tissues compared to normal tissues. The expression of Regulator of chromatin condensation 1/small nucleolar RNA host gene 3/small nucleolar RNA host gene12 in BRCA, LGG and LIHC was associated with TP53 mutations. In addition, Regulator of chromatin condensation 1/small nucleolar RNA host gene 3/small nucleolar RNA host gene12 expression was closely associated with the prognosis of patients with multiple tumors. Immunocorrelation analysis indicated that Regulator of chromatin condensation 1/small nucleolar RNA host gene 3/small nucleolar RNA host gene12 showed a correlation with multiple immune cell infiltration. The results of enrichment analysis suggested that Regulator of chromatin condensation 1/small nucleolar RNA host gene 3/small nucleolar RNA host gene12 was involved in the regulation of cell cycle, apoptosis and other pathways. We found that these effects were mainly mediated by Regulator of chromatin condensation 1, while the trend of small nucleolar RNA host gene 3/small nucleolar RNA host gene12 regulation was also consistent with regulator of chromatin condensation 1. The important role played by Regulator of chromatin condensation 1 in tumor diseases was further corroborated by the study of adjacent lncRNAs.These findings provide new and comprehensive insights into the role of Regulator of chromatin condensation 1/small nucleolar RNA host gene 3/small nucleolar RNA host gene12 in tumor development and show their potential as clinical monitoring and therapy.

## Introduction

With the continuous development of medical treatment, people’s quality of life has been improved and their life expectancy has been extended. Despite the application of various new technologies and methods to the diagnosis and treatment of cancer, the global incidence of new cancer and mortality rates are still high, and the incidence of many cancers is still on the rise, and cancer is one of the leading causes of death worldwide (Vineis and Wild, 2014; Bray et al., 2021). Overall, the global burden of cancer incidence and mortality is rapidly increasing, due to changes in demographic and environmental factors on the one hand, and economic and social influences on the other (Siegel et al., 2021; Sung et al., 2021). According to the latest data from the World Health Organization (WHO) (https://gco.iarc.fr/, accessed on 20 January 2022), cancer with the highest number of incidences as of 2020 is breast cancer, followed by lung and colorectal cancers, and the highest number of deaths is lung cancer.

Studies have shown that alterations in many genes and proteins play a very important role in tumor development, such as TP53 and BRCA1 (Boddicker et al., 2021; Pan et al., 2022). The protein encoded by the Regulator of Chromatin Condensation 1 (RCC1) gene is also known as Cell cycle regulatory protein or Chromosome condensation protein 1. RCC1 is involved in various cellular processes, such as nuclear membrane formation, nucleoplasmic transport and spindle formation. The current study shows that RCC1 is also involved in cell cycle regulation and functions as a guanine nucleotide exchange factor for the intranuclear Ras-like G protein Ran (Dasso et al., 1992; Bischoff and Ponstingl, 1995; Ren et al., 2020). Ran regulates the nuclear-cytoplasmic transport of molecules and regulates the cell cycle by regulating microtubule polymerization and mitotic spindle formation. In these processes, RCC1 is necessary for the coordination of mitosis because of its specific role (Avis and Clarke, 1996; Yudin and Fainzilber, 2009; Boudhraa et al., 2020). Meanwhile, it has been reported that overexpression of RCC1 in normal cells increased Ran⋅GTP levels and accelerated cell cycle and DNA damage repair (Moore, 2001; Cekan et al., 2016). Based on this property, a growing number of experiments have shown that RCC1 is aberrantly expressed in many diseases and plays a regulatory role in cancer progression. It has been shown that RCC1 was identified as a candidate breast cancer susceptibility gene in the Tunisian population by exome sequencing and case-control analysis (Riahi et al., 2018). Other trials have found a strong and significant association between RCC1 expression levels and survival in patients with Colorectal Liver Oligometastases (Deng et al., 2021). In a study of non-small cell lung cancer, knockdown of RCC1 not only significantly inhibited the proliferation of cancer cells but also reduced the volume and weight of tumor models after PD-L1 monoclonal antibody treatment (Zeng et al., 2021).

Both small nucleolar RNA host gene 3 (SNHG3) and small nucleolar RNA host gene 12 (SNHG12) are long non-coding RNAs, which partially overlap and are very close to the sequence of RCC1 on the chromosome. In the analysis of RCC1, we found that the three are highly consistent in pan-cancer, including expression levels, survival, and participation in cellular processes. Recent studies have shown that SNHG3 and SNHG12 are dysregulated in a variety of cancers. SNHG3 and SNHG12 expression were higher in a variety of tumors compared to normal tissues. Furthermore, overexpression of SNHG3 and SNHG12 significantly promoted tumor proliferation, migration and invasion, suggesting that they are an oncogenic lncRNA (Tamang et al., 2019; Xu et al., 2020; Zhang et al., 2020). In breast cancer, SNHG3 functions as a miRNA sponge to promote cancer cell growth and migration (Li et al., 2020; Ma et al., 2020; Wan et al., 2021). Similarly, SNHG12 promotes proliferation and inhibits apoptosis in triple-negative breast cancer cells (Wang O. et al., 2017). In osteosarcoma, SNHG3 regulates cell migration and invasion through the miRNA-151a-3p/RAB22A axis, promotes cell growth by sponging miR-196a-5p and indicates poor survival (Chen J. et al., 2019; Zheng et al., 2019). At the same time, SNHG12 promotes tumorigenesis and metastasis in osteosarcoma through the miR-195–5p/Notch2 axis and mediates resistance to doxorubicin through the miR-320a/MCL1 axis (Zhou B. et al., 2018; Zhou S. et al., 2018). Similar situations are shown in hepatocellular carcinoma (Hou et al., 2011; Lan et al., 2017; Wu et al., 2019; Zhang P. F. et al., 2019; Zhao et al., 2019), renal cell carcinoma (Chen Q. et al., 2019; Zhang C. et al., 2019), bladder cancer (Jiang et al., 2018; Dai et al., 2020), colorectal cancer (Huang et al., 2017; Wang J. Z. et al., 2017; Dacheng et al., 2020), etc.

In the analysis of RCC1, SNHG3 and SNHG12 we found that their expression was upregulated in many tumors. Also, their genomic alterations were very consistent due to their very close location on the chromosome. As the study progressed, they also showed similarities in survival, prognosis, immune regulation, and cellular processes. Overall, this study aims to provide a comprehensive analysis of the three to understand their possible common mechanisms in tumor diseases and to provide new ideas for future studies.

## Result

### Regulator of chromatin condensation 1/small nucleolar RNA host gene 3/Small nucleolar RNA host gene 12 expressions are upregulated in multiple cancers

**FIGURE 1:**
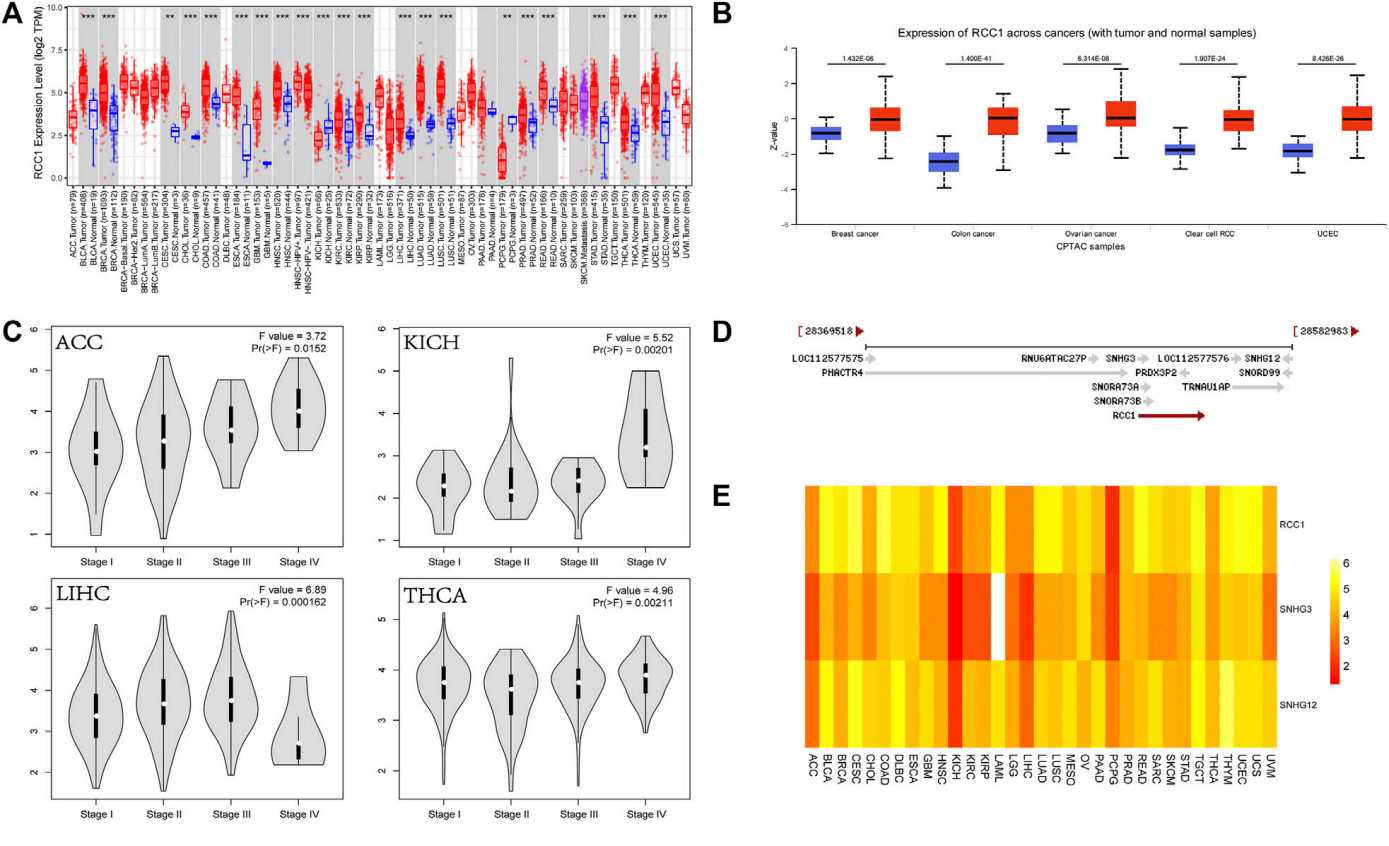
Expression of RCC1 and correlation of RCC1/SNHG3/SNHG12 in pan-cancer. **(A)** RCC1 expression in pan-cancer. **(B)** Protein expression levels of RCC1 in selected cancers. **(C)** Differential expression of RCC1 in ACC/KICH/LIHC/THCA in staging. **(D)** Relationship of RCC1/SNHG3/SNHG12 on chromosomal location. **(E)** Comparison of RCC1/SNHG3/SNHG12 expression in pan-cancer. **p < 0.01; ***p < 0.001.

The analysis of TCGA and CPTAC data showed that RCC1 expression was significantly upregulated in a variety of cancers (Figures 1A,B), and was higher in some cancer stage III and IV patient samples than in stage I and II (Figure 1C). RCC1 partially overlaps with SNHG3 at the chromosomal location and is very close to SNHG12 (Figure 1D). Their expression in pan-cancer was significantly positively correlated (Figure 1E).

**FIGURE 2:**
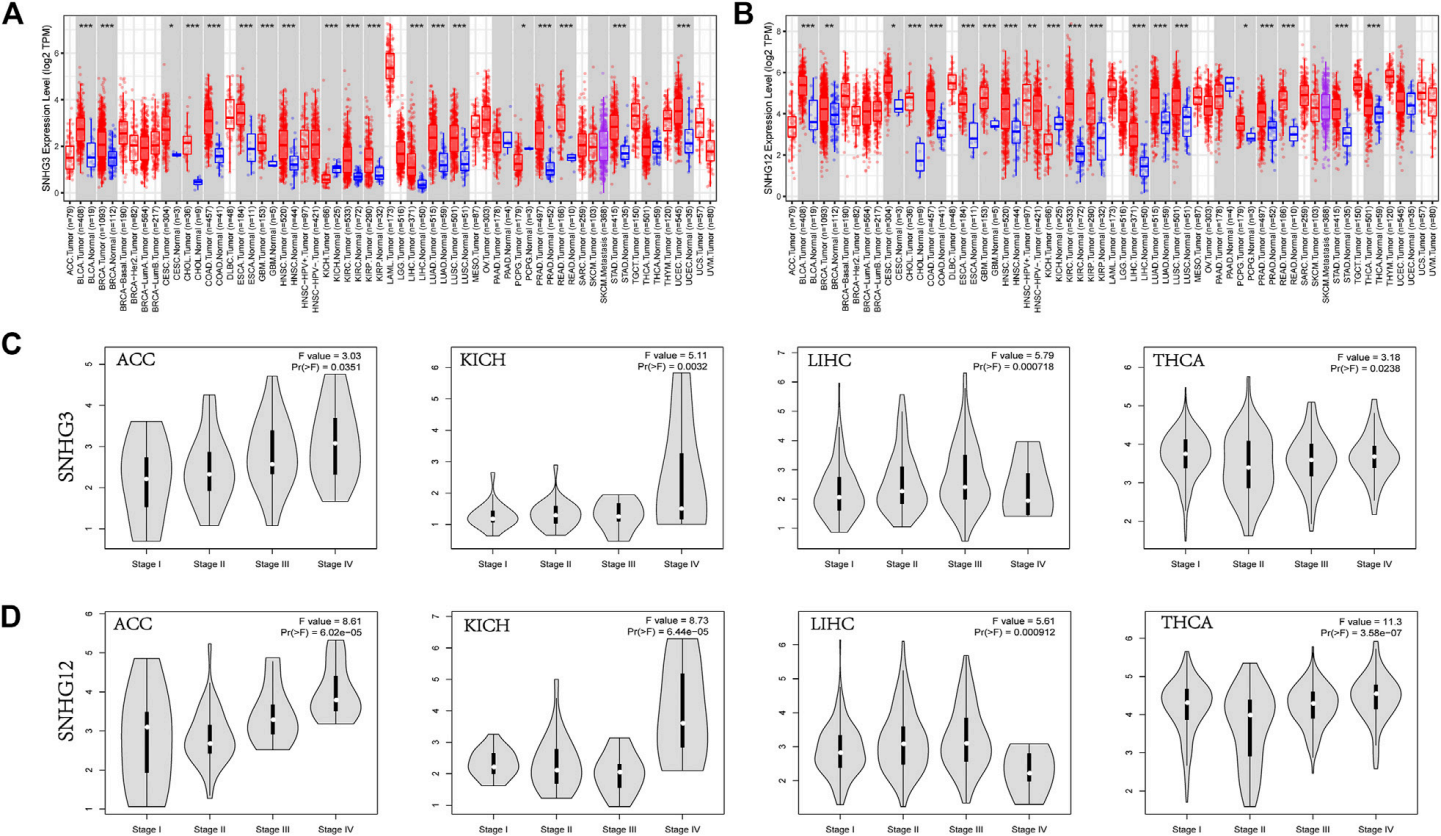
SNHG3/SNHG12 expression in pan-cancer. **(A,B)** SNHG3 and SNHG12 expression in pan-cancer. **(C,D)** Differential expression of SNHG3 and SNHG12 in different stages in ACC/KICH/LIHC/THCA. *p < 0.05; **p < 0.01; ***p < 0.001.

The expression of SNHG3 and SNHG12 in pan-cancer showed high similarity with the expression of RCC1 (Figures 2A,B). And their expression were higher in stages III and IV than in stages I and II in patients with ACC, KICH, LIHC, and THCA (Figures 2C,D).

### Analysis of Regulator of chromatin condensation 1/small nucleolar RNA host gene 3/Small nucleolar RNA host gene 12 gene alterations

**FIGURE 3:**
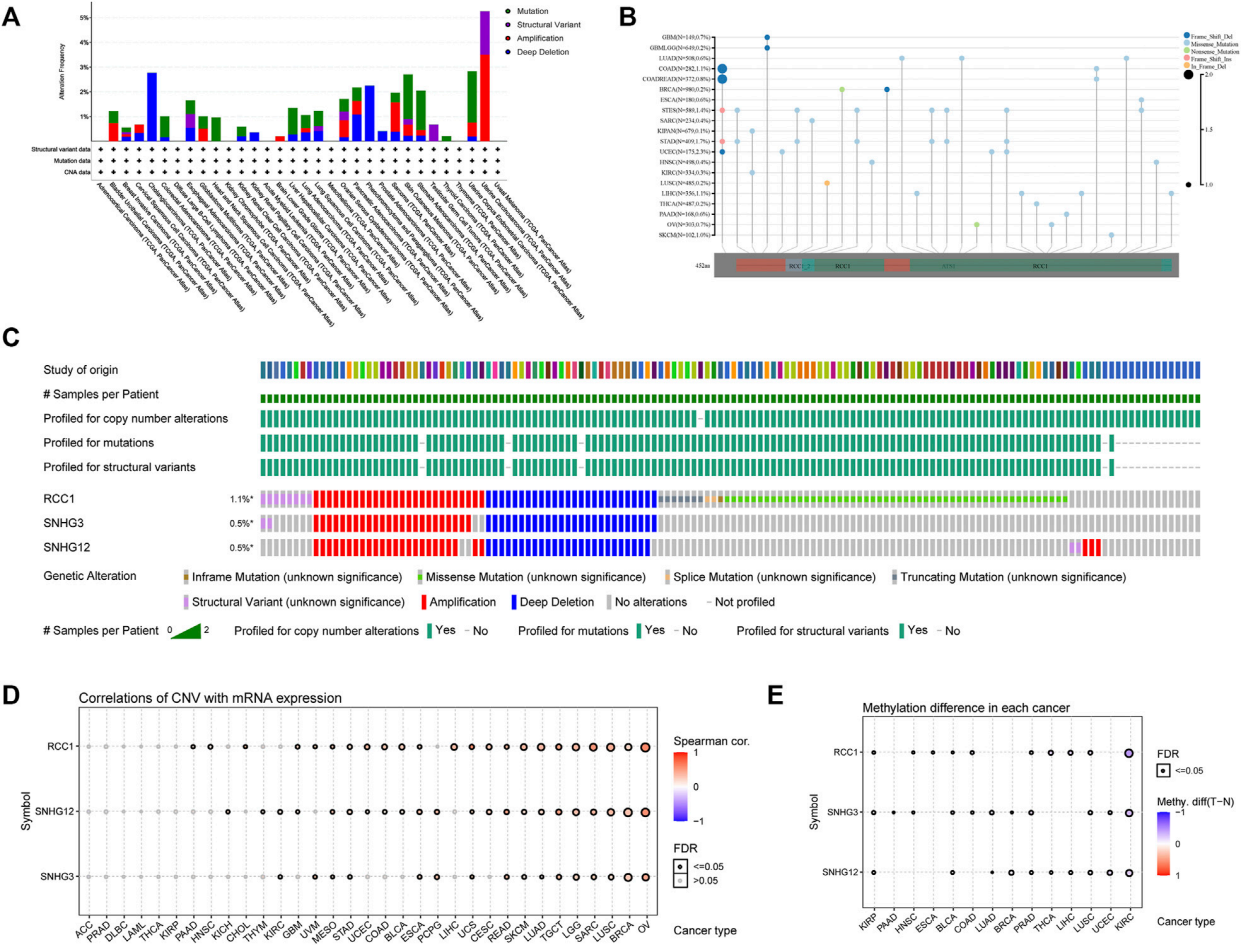
Genetic alternation of RCC1/SNHG3/SNHG12 in different tumors. **(A)** RCC1 mutation frequency in multiple TCGA pan-cancer studies. **(B)** Mutation diagram of RCC1 in different cancer types across protein domains. **(C)** Genome alternation of RCC1, SNHG3 and SNHG12. **(D)** Correlation of CNV with mRNA expression. **(E)** Methylation difference in each cancer.

The RCC1 gene alterations were analyzed in the TCGA cohort, and the highest frequency of RCC1 gene alterations (>5%), mainly copy number amplification, was found in UCS patients (Figure 3A). The overall type and location of RCC1 gene alterations were analyzed, and missense mutations were found to be the main type of RCC1 gene alterations (Figure 3B). RCC1/SNHG3/SNHG12 are mostly the same samples in the TCGA cohort showing genetic alterations due to their proximity on the chromosome (Figure 3C). Overall, their CNV was positively correlated with gene expression in a variety of tumors. (Figure 3D). However, their methylation levels were less consistent (Figure 3E). Interestingly, SNHG3 showed promoter hypermethylation levels in tumor tissues. While this phenomenon was absent in SNHG12 (Supplementary Figure S1).

In addition, the mutation profile of TP53 showed highly significant differences in the RCC1/SNHG3/SNHG12 low and high expression samples of BRCA, LGG, and LIHC (Supplementary Figure S2). The expression levels of RCC1/SNHG3/SNHG12 in BRCA, LGG, and LIHC were found to be correlated with TP53 mutations by comparing the differential gene expression between different mutation statuses of TP53 through the TIMER2.0 database. This suggests that TP53 mutations may affect the gene expression of RCC1/SNHG3/SNHG12 (Supplementary Figure S3).

### Tumor mutation load and microsatellite deletion analysis of regulator of chromatin condensation 1/small nucleolar RNA host gene 3/small nucleolar RNA host gene 12

**FIGURE 4:**
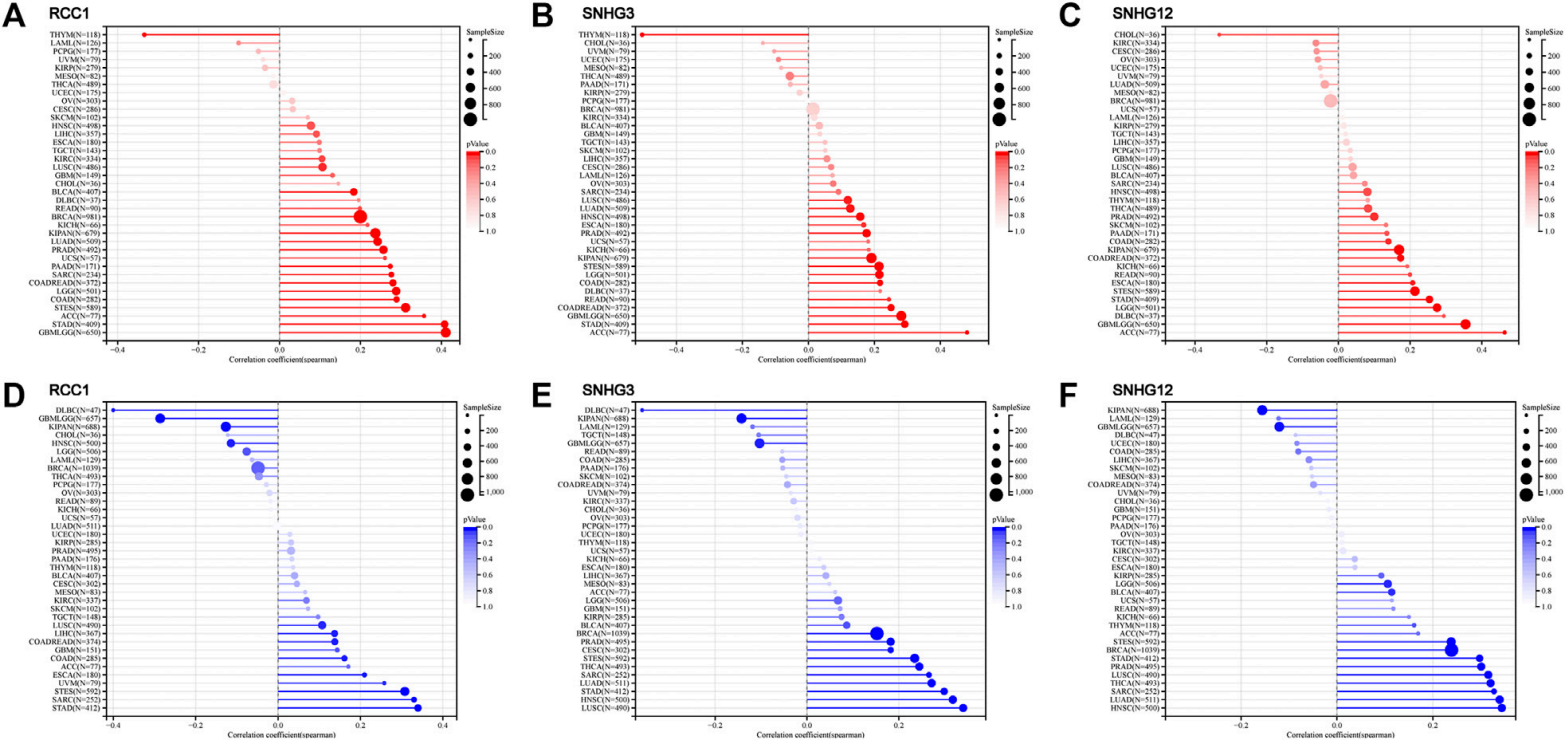
Correlation between RCC1, SNHG3, SNHG12 expression and TMB/MSI in cancers. **(A–C)** TMB. **(D–F)** MSI.

TMB is the number of somatic mutations in the tumor genome after removal of germline mutations. Higher TMB indicates that the more neoantigens the tumor produces, the more easily the tumor is recognized by immune cells. After analyzing the relationship between RCC1 expression and TMB in the TCGA cohort, RCC1 expression in GBMLGG, LGG, LUAD, COAD, COADREAD, BRCA, STES, SARC, KIPAN, STAD, PRAD, LUSC, PAAD, BLCA, and ACC were found to be positively correlated with TMB. Overall, the expression of RCC1/SNHG3/SNHG12 was positively correlated with TMB in GBMLGG, LGG, COAD, COADREAD, STES, KIPAN, STAD, PRAD, and ACC (Figures 4A–C).

MCI, also known as short tandem repeats, is an inherited mutational state caused by defects in DNA mismatch repair function. Similarly, in the analysis of RCC1 expression about MSI in the TCGA cohort, we found that RCC1 expression in COAD, COADREAD, ESCA, STES, SARC, STAD, LUSC, LIHC, and UVM was positively correlated with MSI. The tumors with a positive correlation between RCC1/SNHG3/SNHG12 expression and MSI included STES, SARC, STAD and LUSC, and those with negative correlation with MSI included GBMLGG and KIPAN (Figures 4D–F).

### Regulator of chromatin condensation 1/small nucleolar RNA host gene 3/Small nucleolar RNA host gene 12 survival-related analysis

**FIGURE 5:**
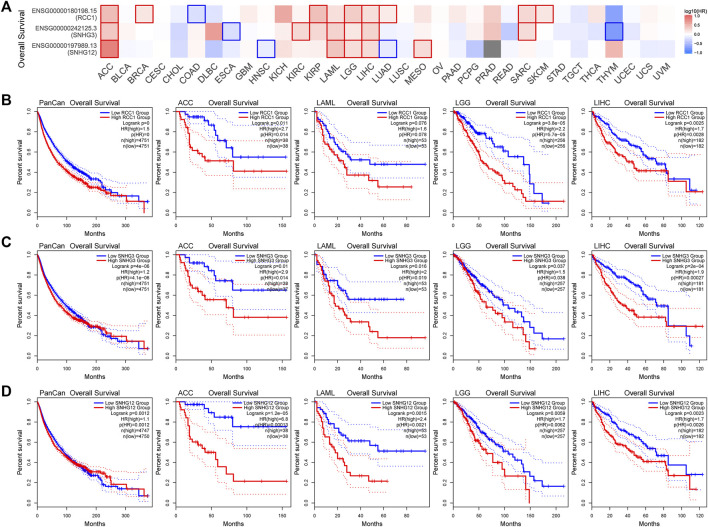
Correlation between RCC1/SNHG3/SNHG12 expression and overall survival of tumors. **(A)** Survival map of the correlation between the expression of RCC1/SNHG3/SNHG12 and the overall survival of patients in different tumors. **(B–D)** K-M plot of overall survival of PANCAN/ACC/LAML/LGG/LIHC.

**FIGURE 6:**
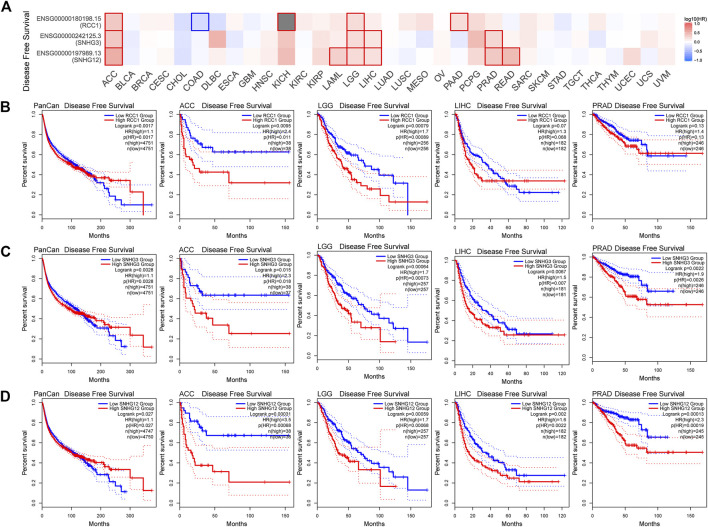
Correlation between RCC1/SNHG3/SNHG12 expression and disease-free survival of tumors. **(A)** Survival map of the correlation between the expression of RCC1/SNHG3/SNHG12 and the disease-free survival of patients in different tumors. **(B–D)** K-M plot of disease-free survival of PANCAN/ACC/LGG/LIHC/PRAD.

Overall, the expression levels of RCC1/SNHG3/SNHG12 in the TCGA data correlated with overall survival (Figure 5A). Patients with high RCC1/SNHG3/SNHG12 expression in pan-cancer have poor early OS. Also high expression of RCC1/SNHG3/SNHG12 correlated with low OS of ACC, LAML, LGG, and LIHC. High expression of RCC1/SNHG3/SNHG12 is a poor risk factor for OS (Figures 5B–D). This phenomenon is similar in DFS (Figure 6A). High expression of RCC1/SNHG3/SNHG12 was poor in early DFS in pan-cancer and correlated with low DFS in ACC, LGG, LIHC, and PRAD (Figures 6B–D).

### Regulator of chromatin condensation 1/small nucleolar RNA host gene 3/Small nucleolar RNA host gene 12 immune-related analysis

**FIGURE 7:**
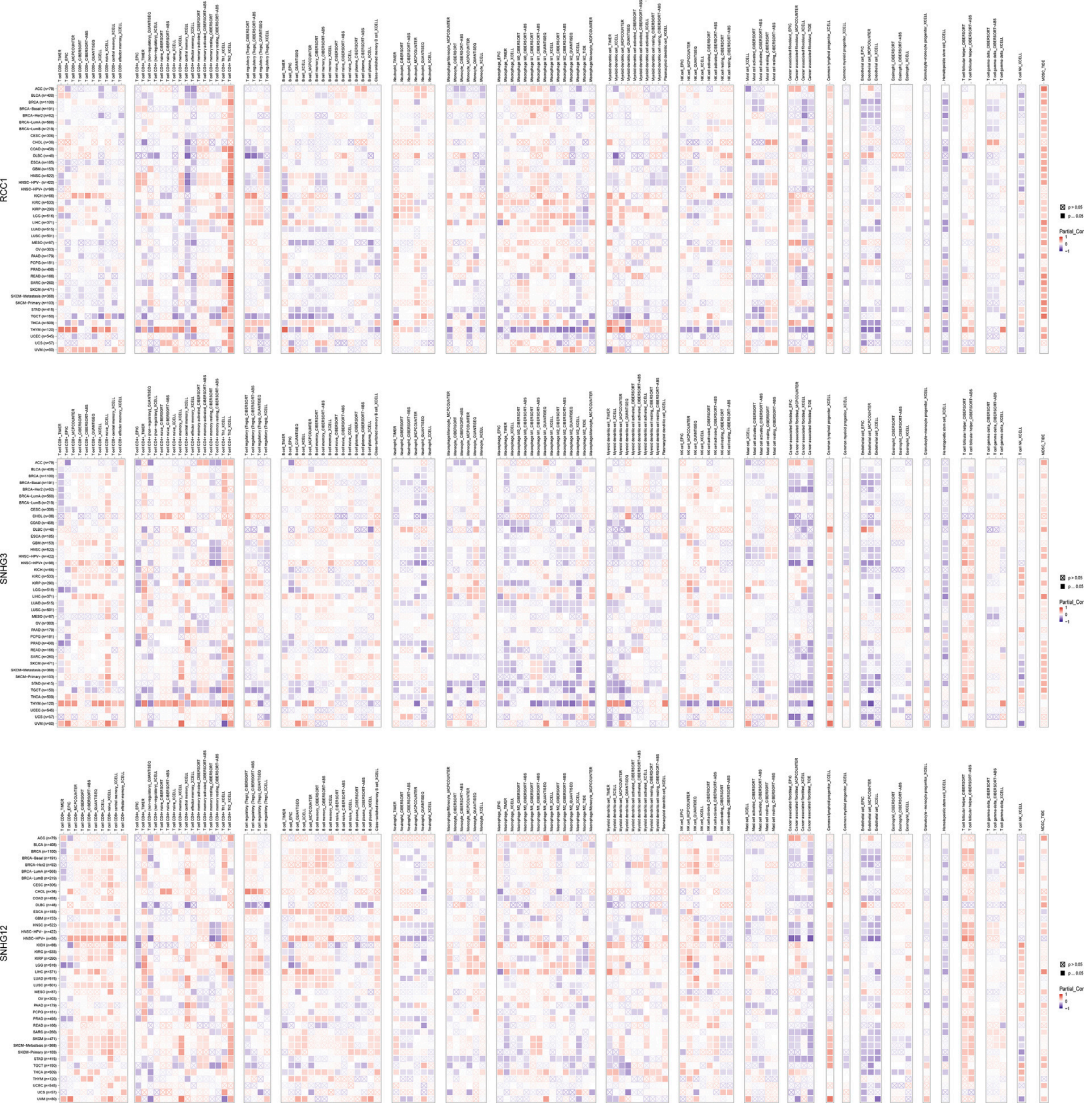
Correlation of RCC1/SNHG3/SNHG12 expression and immune infiltration in cancers.

**FIGURE 8:**
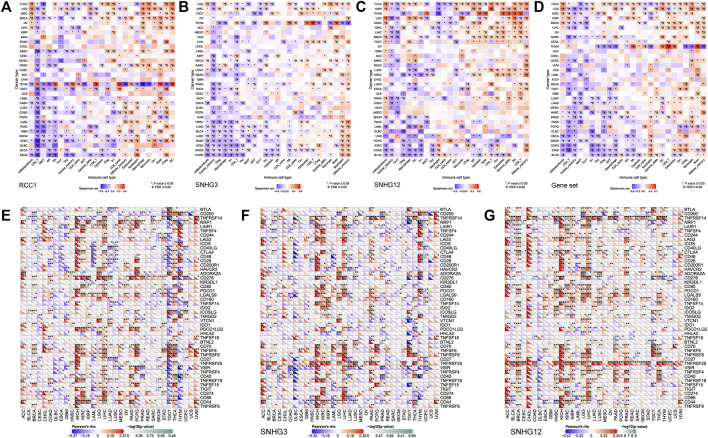
Association between RCC1/SNHG3/SNHG12 expression and immune infiltration and immune checkpoint genes expression in cancers. **(A–D)** Correlation of GSVA score with immune infiltration in different cancers. **(E–G)** Correlation of mRNA expression with immune checkpoint genes expression in different cancers.

The landscape of RCC1/SNHG3/SNHG12 associated with various immune infiltrations in human cancers was demonstrated using TIMER2.0. Overall, RCC1 and SNHG3 showed concordance in BRCA, HNSC, KIRC, KIRP, LGG, LIHC, STAD, TGCT, THYM, while SNHG12 showed similarity with them only in HNSC, LIHC. Among them, RCC1/SNHG3/SNHG12 was negatively correlated with the level of immune infiltration of various infiltrating cells such as endothelial cells and hematopoietic stem cells but positively correlated with the abundance of myeloid-derived suppressor cells (MDSC) (Figure 7). The results suggest that RCC1/SNHG3/SNHG12 are involved in the immune infiltration process to some extent and play an important role in immune-tumor interactions. Notably, they both showed a strong correlation with immune cell infiltration in THYM, suggesting their likely involvement in the THYM process and their potential as targeted therapeutic targets (Figures 8A–D).

The pan-cancer correlations between RCC1/SNHG3/SNHG12 and immune checkpoints were displayed (Figures 8E–G). The expression of RCC1/SNHG3/SNHG12 in ACC, KICH, and LIHC showed a significant positive correlation with most of the immune checkpoint genes. The expression of RCC1 in PCPG showed a positive correlation with many immune checkpoint genes but a partial negative correlation in TGCT, THYM. Meanwhile, RCC1 expression was positively correlated with CD276 in different tumors. The expression of SNHG3/SNHG12 in different tumors showed a positive correlation with TNFRSF25. In addition, SNHG12 expression also showed a significant positive correlation with TNFRSF14.

### Regulator of chromatin condensation 1/small nucleolar RNA host gene 3/Small nucleolar RNA host gene 12 involved in cell cycle and apoptosis regulation

**FIGURE 9:**
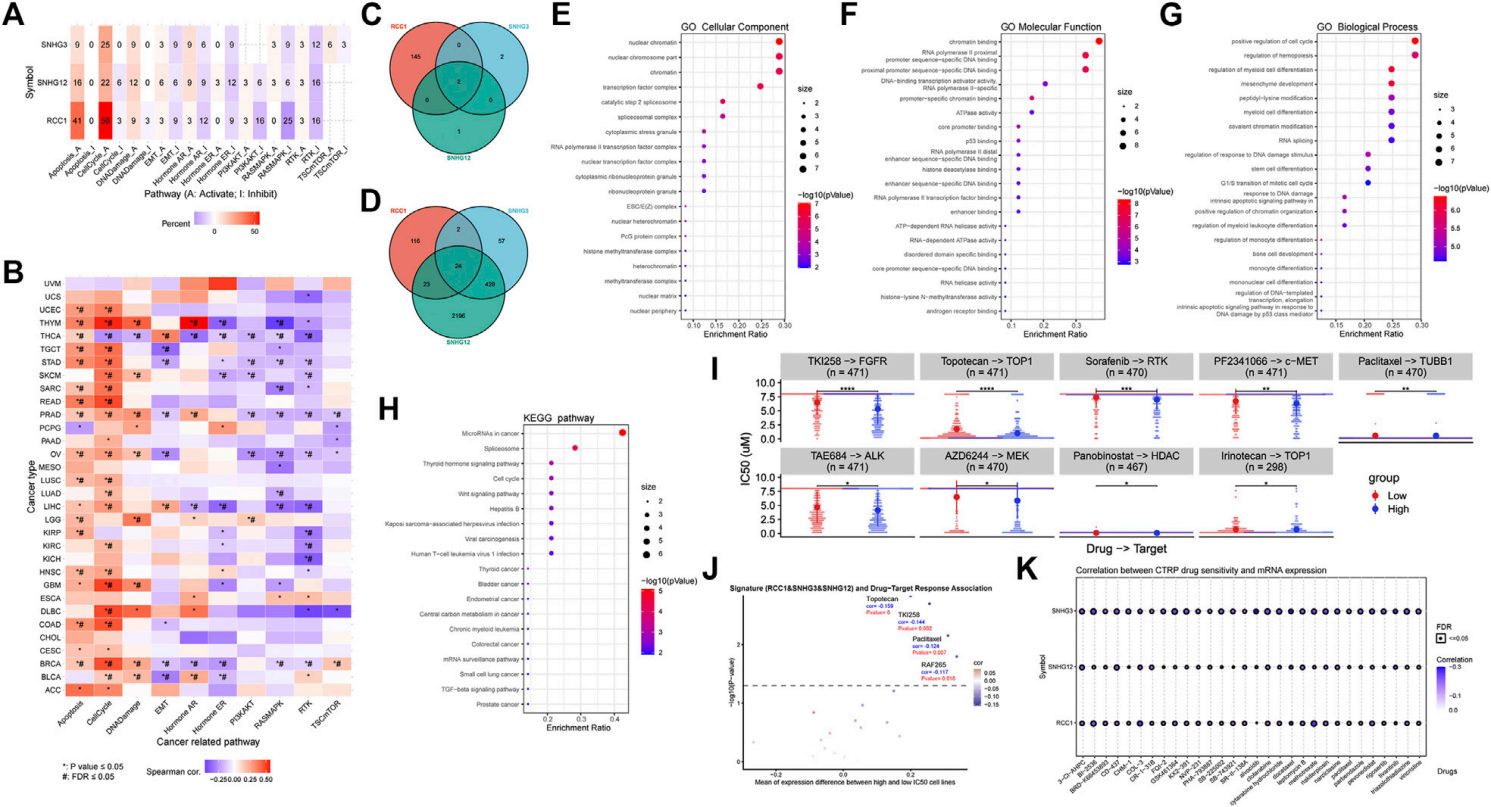
RCC1/SNHG3/SNHG12 is involved in cell cycle apoptosis regulation. **(A,B)** RCC1/SNHG3/SNHG12 as a single gene or gene set level analysis for expression and pathway activity. Numbers represent the percentage of cancers in which specific gene’s mRNA expression has a potential effect on pathway activity. **(C,D)** Intersection analysis of the RCC1/SNHG3/SNHG12interacted or correlated genes. 24 related genes were identified in the latter. **(E–H)** GO and KEGG analysis of 24 related genes. **(I,J)** RCC1/SNHG3/SNHG12 as a signature for drug-target response difference and association in pan-cancer. **(K)** Correlation between CTRP drug sensitivity and mRNA expression.

In general, the effects and regulation of RCC1/SNHG3/SNHG12 on different cellular activities were generally consistent, especially in the positive regulation of apoptosis and cell cycle and the negative regulation of the RTK pathway. In addition, significant differences were shown in the activities of each cell in BRCA, LIHC, PRAD, THCA, THYM and other cancers, suggesting their possible more critical regulatory roles in these cancers (Figures 9A,B). Based on the BioGRID database summary, we found that tripartite motif containing 25 (TRIM25)、heterogeneous nuclear ribonucleoprotein H1(HNRNPH1) are in their intersection. TRIM25 is an RNA binding protein, functions as a ubiquitin E3 ligase, and is involved in multiple cellular processes. HNRNPH1 is a component of the heterogeneous nuclear ribonucleoprotein (hnRNP) complexes which provide the substrate for the processing events that pre-mRNAs undergo before becoming functional, translatable mRNAs in the cytoplasm (Figure 9C). After aggregating the search results of proteins that can interact with RCC1/SNHG3/SNHG12 from multiple databases, we obtained a total of 24 proteins by taking intersections. After GO and KEGG analysis, the result showed that cell cycle and pre-mRNA processing were significantly enriched (Figures 9D–H). Similarly, we performed GO and KEGG analysis using David, and the results remained consistent (Supplementary Table S1).

In the analysis RCC1/SNHG3/SNHG12 as a signature for drug-target response difference and association in pan-cancer, Topotecan, TKI258 and Paclitaxal were found which showed significant differences in different expression levels (Figures 9I,J). At the same time, Paclitaxal also showed differences in the summary of the correlation between gene expression and the sensitivity of CTRP drugs (top 30) in pan-cancer (Figure 9K).

## Discussion

It is now known that RCC1 is involved in the regulation of cell cycle processes and can be involved in the repair of DNA damage. Also, there have been many reports indicating that SNHG3 and SNHG12 are aberrantly expressed in many tumor tissues and can be involved in the development of a variety of tumors and diseases. We evaluated the expression of RCC1/SNHG3/SNHG12 in 33 different cancer types using several databases such as GEPIA, UALCAN, and TIMER2.0, and found that they were significantly differentially expressed in tumor tissues and normal tissues. The results showed that, overall, they were all upregulated in BLCA, BRCA, CESC, CHOL, COAD, ESCA, GBM, HNSC, KIRC, KIRP, LIHC, LUAD, LUSC, PRAD, READ, and STAD tumor tissues, while downregulated in KICH. In addition, RCC1 and SNHG12 expression were upregulated in THCA, and RCC1 and SNHG3 expression were upregulated in UCEC. However, RCC1 and SNHG3 expression were downregulated in PCPG tumors, while SNHG12 showed upregulation. However, it is noteworthy that the number of normal samples relative to PCPG was only three and the expression differences were not significant.

Regarding the reason for the downregulation of RCC1/SNHG3/SNHG12 expression in KICH, the methylation of RCC1/SNHG3/SNHG12 genes was first analyzed by MEXPRESS tool (https://mexpress.be/). The results showed no statistical significance (Supplementary Figure S4A). Next, we analyzed the tumor mutation landscape of RCC1/SNHG3/SNHG12 expression in KICH using the SangerBox tool. However, limited by the sample size and other factors, there was no significant difference in the mutation landscape of each gene under high or low expression of RCC1/SNHG3/SNHG12 (Supplementary Figure S4B). Subsequently, we analyzed the overall differential genes in KICH tumors and their chromosomal distribution using the GEPIA2 tool. It was found that the differential genes were mostly altered with downregulated expression (Supplementary Figure S4C). We then pooled all differential genes in BLCA, BRCA, CESC, COAD, ESCA, GBM, HNSC, KIRC, KIRP, LIHC, LUAD, LUSC, PRAD, READ, STAD, and KICH. The genes were divided into two groups according to whether they were downregulated in KICH and upregulated in the rest of the cancers or upregulated in KICH and downregulated in the rest of the cancers. In each group, the intersection of the differential genes in each cancer was taken. However, neither group was finally enriched for the key genes (Supplementary Figure S4D,E). Since KICH belongs to a rare type of kidney cancer, there are not many studies and analyses about it. Here, we speculate that it may be the result of the synergistic regulation of multiple genes.

In terms of genetic variation, since RCC1/SNHG3/SNHG12 are located adjacent to each other on the chromosome, their CNV and methylation levels are more similar. Especially, in OV, BRCA, LUSC, SARC, LGG and other tumors, their CNV and mRNA expression showed a very significant positive correlation. The effect of methylation level on the mRNA expression of SNHG3 and SNHG12 was greater than that of RCC1. This result may be due to the different lengths of the genes.

RCC1/SNHG3/SNHG12 are not only highly expressed in a variety of cancers, but also are risk factors for poor prognosis. Overall, RCC1/SNHG3/SNHG12 can be used as a marker of poor prognosis in the early stages of pan-cancer. High expression of RCC1/SNHG3/SNHG12 in patients with a variety of cancers including ACC, KIRP, LAML, LGG, LIHC, and PRAD predicted lower overall survival and disease free survival. We additionally analyzed the OS and DFS of RCC1/SNHG3/SNHG12 in KICH. The results showed a trend towards lower survival in patients with high expression of RCC1/SNHG3/SNHG12. This result was statistically different in RCC1, but not statistically significant in SNHG3 and SNHG12 (Supplementary Figure S5).

In the next immune correlation analysis, we found some very interesting phenomena. Based on the results of the TIMER 2.0 tool, it appears that RCC1/SNHG3/SNHG12 were positively correlated with immune cell infiltration in MDSC and negatively correlated with hematopoietic stem cell and eosinophil. In addition, RCC1 was positively correlated with the degree of infiltration of common lymphoid progenitor. MDSC can significantly suppress the immune cell response, protect cancer from the immune system, and makes the tumor resistant to immunotherapy (Tesi, 2019; Hegde et al., 2021). T cell follicular helper (Tfh) is a specific subpopulation of CD4^+^ T cells that plays a key role in protective immunity. Tfh function is dysregulated in several diseases where antibody production is excessive or insufficient. The amount of circulating Tfh is increased in the blood of patients with autoimmune diseases (Crotty, 2011; Hirahara and Nakayama, 2016; Read et al., 2019; Song and Craft, 2019). We think that SNHG3/SNHG12 is positively correlated with immune infiltration in MDSC on the one hand, and with Tfh on the other hand, suggesting that the immune regulatory processes involved are complex. Meanwhile, combined with the results of survival analysis, the role of RCC1/SNHG3/SNHG12 in some cancers is dominated by suppression of immune response.

In contrast, for individual cancers, SNHG3 behaved more similarly to RCC1, especially in LGG, LIHC, PCPG, THYM, etc. In THYM, RCC1/SNHG3 was positively correlated with immune infiltration of CD8^+^ and CD4^+^ T cells and negatively correlated with immune infiltration of macrophage, NK cells, and cancer associated fibroblast (CAF). CAF is an important component of the tumor microenvironment and has multiple functions including matrix remodeling. Current studies suggest that CAF drives cancer growth and progression by remodeling the tumor microenvironment and contributes to increased tumor drug resistance (Richards et al., 2017; Shan et al., 2017; Sahai et al., 2020; Vaquero et al., 2020). We additionally analyzed the OS and DFS of RCC1/SNHG3/SNHG12 in THYM patients. The results showed that the expression of RCC1/SNHG3 correlated with OS in THYM patients, and the OS was higher in patients with high expression of RCC1/SNHG3. In contrast, this phenomenon was not mentioned in SNHG12 (Supplementary Figure S6).

The results of immunoassays based on the GSCA tool showed that SNHG3 behaved more similarly to RCC1, while there were some differences in SNHG12. Similarly, RCC1/SNHG3/SNHG12 showed a very significant correlation with various immune cell infiltration in THYM. When RCC1/SNHG3/SNHG12 was analyzed as a gene set, its overall performance was very similar to that of RCC1. We suggest that the gene encoding the protein plays a primary role in the process of immune regulation, while the lncRNA may play a secondary supporting role. Taken together, RCC1/SNHG3/SNHG12 are involved in the process of immune cell infiltration, and the process is more complex. In particular, RCC1 and SNHG3 can be used as immune detection markers for THYM.

The phenomenon of RCC1/SNHG3/SNHG12 in immune regulation is also reflected in the regulation of cellular processes. In particular, the correlation of RCC1 was more significant in apoptosis and cell cycle. The correlation of SNHG3/SNHG12 was not as significant as that of RCC1, but the regulatory trend remained consistent with RCC1. In addition, the effects of RCC1/SNHG3/SNHG12 on each cellular pathway in BRCA, PRAD, THCA, and THYM were significantly correlated, especially in THYM. Notably, in addition to the involvement of RCC1 in DNA Damage Response, Cell Cycle, and Apoptosis pathways that are currently known, RCC1/SNHG3/SNHG12 also positively correlated with hormone AR pathway and negatively correlated with hormone ER in THYM. Androgen receptor (AR) belongs to the steroid hormone family and is involved in the regulation of normal growth and development of various target organs. The current research and application of AR are mainly in prostate cancer and breast cancer (Heinlein and Chang, 2002; Li et al., 2019). Several studies have been reported on the involvement of LncRNA in the regulation of AR (Collina et al., 2019; Kumar et al., 2021). In Philling et al. study, activation of AR increased cell viability and survival and attenuated G2/M arrest. AR negatively regulated spindle checkpoint signaling, leading to premature mitotic progression and apoptotic cell death evasion (Pilling et al., 2022). Estrogen receptors (ER) belong to protein molecules, including nuclear and membranous receptors (Eyster, 2016). Most reports on ER have focused on breast cancer diagnosis and treatment (Bai and Gust, 2009; Yip and Rhodes, 2014). In bladder cancer, ERα activation is thought to have an inhibitory role in tumor growth, as its knockdown promotes the growth of cancer cells and xenograft tumors (Hsu et al., 2014). And in a study of gastric cancer, the authors found that ERα overexpression significantly inhibited cell growth and proliferation, promoted apoptosis, and blocked cell entry into the G1/G0 phase (Zhou et al., 2013; Ur Rahman and Cao, 2016). Thus, it seems reasonable that RCC1/SNHG3/SNHG12 are positively correlated with the AR pathway and negatively correlated with the ER in THYM. On the one hand, they positively regulate the AR pathway to attenuate G2/M arrest and promote the mitotic process. On the other hand, they negatively regulate the ER pathway to ensure that cells enter the G1/G0 phase and ensure the stability of the mitotic process. The overall trend is consistent with the regulation of cell cycle and DNA damage repair by RCC1.

After GO and KEGG analysis of the proteins that can interact with RCC1/SNHG3/SNHG12, we found that the cellular components and biological processes they are involved in are very similar to the localization and function of RCC1. GO analysis showed that they are mostly located on chromatin in the nucleus and their molecular functions are mainly involved in chromatin binding, DNA binding, p53 binding, etc. Remarkably, in addition to the positive regulation of cell cycle and mitosis, they are also involved in the regulation of a variety of cells and their differentiation in terms of biological processes, including hemopoiesis, myeloid cell, stem cell, myeloid leukocyte, etc. The positive correlation of RCC1/SNHG3/SNHG12 with immune cell infiltration in MDSC seems to be more convincing in the previous immune correlation analysis. In a subset of tumors, high expression of RCC1/SNHG3/SNHG12 caused enhanced mitotic and DNA damage repair processes in tumor cells, thereby enhancing cell viability and promoting cell proliferation. At the same time, RCC1/SNHG3/SNHG12 may in turn influence the regulation of immune cell infiltration in this tumor by directly or indirectly regulating the proliferation and differentiation of some immune cells.

Besides, we observed that KEGG analysis significantly enriched to MicroRNAs in cancer pathway. The microRNAs involved include let-7c, miR-1, miR-21,miR-30, miR-125, miR-143, miR-194, miR-223 (Supplementary Figure S7). As we know, there have been many studies showing that miRNAs play a very important role in the development of tumor diseases and can be used as indicators of cancer diagnosis and prognosis. Among them, let-7, miR-21, and miR-30 have shown strong biological effects in a variety of tumors, including but not limited to regulation of cell proliferation, modulation of tumor metastasis, and regulation of angiogenesis. The cascade effects are induced by silencing specific target genes (Lee and Dutta, 2009). In addition, the Spliceosome and Thyroid hormone signaling pathway were involved in the results. The above results indicate that RCC1/SNHG3/SNHG12 are not only involved in the regulation of cell cycle and apoptosis, but also extensively affect the epigenetic regulatory processes. That is, they have a broad and profound impact on tumor growth and metastasis by directly participating in the regulation of the cell cycle and indirectly regulating gene expression, mRNA synthesis and processing, and so on. These processes and pathways deserve further study.

LncRNAs can function as cis-acting elements. RCC1 shows strong similarity to SNHG3/SNHG12 expression and action. This also suggests that RCC1 plays an important role as a potential biomarker in tumor diseases. We believe that adding the analysis of its neighboring lncRNAs to the study of gene expression and action provides a lighter and more convincing picture. The strong tumor correlation shown by RCC1/SNHG3/SNHG12, especially RCC1, in tumor diseases deserves further investigation.

## Method

### Gene expression and protein expression analysis

Differential expression of RCC1/SNHG3/SNHG12 in different tumor tissues and normal tissues was analyzed using the “Exploration” module of the TIMER2 tool (http://timer.cistrome.org/). The “CPTAC” module of the online tool UALCAN (http://ualcan.path.uab.edu/) was used to complement the analysis of the differential protein expression of RCC1 in different tumors. RCC1/SNHG3/SNHG12 positional relationships on chromosomes were based on the results of NCBI (https://www.ncbi.nlm.nih.gov/) searches.

The expression differences of RCC1/SNHG3/SNHG12 in different tumor stages were analyzed by the “Expression DIY” module in the GEPIA2 tool (http://gepia2.cancer-pku.cn/#index). The“log2 (TPM +1)” was used for log-scale in violin plots. In addition, after selecting “Multiple Genes Comparison” in the “Expression DIY” module, we entered RCC1, SNHG3 and SNHG12 genes and analyzed the expression correlation between them. The closer the color of different genes in the same tumor, the more equal the expression level between them. The significance of the differences is indicated in the graphs.

### Genetic variation and DNA methylation analysis

The genetic variants of RCC1/SNHG3/SNHG12 genes in tumors were analyzed using the cBioPortal tool (https://www.cbioportal.org/) based on TCGA PanCancer Atlas Studies to obtain mutation status, mutation frequency and copy number change data. Non-parametric tests (rank sum test) were used for comparison. The genomic alterations of RCC1/SNHG3/SNHG12 in the TCGA cohort were compared by entering the SNHG3/SNHG12 gene information in the “OncoPrint” module.

The mutation landscape of the RCC1 gene was mapped by integrating the mutation data of the samples and obtaining the structural domain information of the protein from the R package maftools (version 2.2.10) using the Sangerbox tool (http://vip.sangerbox.com/home.html). In addition, we also mapped the tumor mutation landscape of RCC1/SNHG3/SNHG12 in BRCA/LGG/LIHC in the “Pancancer Analysis” module.

Also, to evaluate the effect of copy number variation and methylation changes on gene expression, we used the “Mutation” module of the GSCA tool (http://bioinfo.life.hust.edu.cn/GSCA/#/) to evaluate the relationship between RCC1/SNHG3/SNHG12 gene expression levels and CNV and methylation by selecting “CNV and Expression” and “Methylation and Expression” of the GSCA tool to evaluate the correlation between the expression levels of RCC1/SNHG3/SNHG12 genes and CNV and methylation.

In addition, we obtained the methylation levels of SNHG3 and SNHG12 in several tumor tissues and normal tissues using the “Methylation” module of the Lncbook database (https://ngdc.cncb.ac.cn/lncbook/index). The correlation between TP53 and RCC1/SNHG3/SNHG12 expression was analyzed using the “Explore” module of the TIMER2 tool. Because they all showed statistically significant in BRCA/LGG/LIHC tumors, we also explored in detail the differences in RCC1/SNHG3/SNHG12 expression levels in WT and Muted TP53 among them.

Tumor mutation load (TMB) refers to the number of somatic mutations in the tumor genome after removal of germline mutations. Microsatellite deletion (MSI) refers to the inherited mutational status caused by defective DNA mismatch repair function. TMB and MSI of RCC1/SNHG3/SNHG12 genes were analyzed using the “Pancancer Analysis” module of the Sangerbox tool. After integrating the TMB/MSI and gene expression data of the samples, a log2 (x+1) transformation was performed for each expression value, and cancers with less than three samples in a single cancer species were also excluded.

### Survival analysis

Patients were divided into high and low expression groups according to the median RCC1/SNHG3/SNHG12 expression. In this way, the “Survival Analysis” module of the GEPIA2 tool was used to analyze the Overall survival (OS) and Disease free survival (DFS) of all tumors in the TCGA cohort. Meanwhile, we focused on the analysis of OS of RCC1/SNHG3/SNHG12 in pan-cancer, ACC, LAML, LGG, LIHC, and DFS in pan-cancer, ACC, LGG, LIHC, and PRAD. Survival comparison maps and Kaplan–Meier survival curves were obtained. Log-rank *p* value and hazard ratio were calculated.

### Immune-related analysis

To investigate the relationship between gene expression and immune cell infiltration, the relationship between RCC1/SNHG3/SNHG12 and immune infiltration of multiple immune cells was analyzed separately in the “Immune” module using the TIMER2 tool. Meanwhile, using the “Immune” module of the GSCA tool, we selected “Immune infiltration and mRNA expression” and “Immune infiltration and GSVA score” and analyzed the association between individual RCC1/SNHG3/SNHG12 genes and immune cell infiltration, respectively. In addition, after considering them as a gene set, their correlation with immune cell infiltration was analyzed.

Potential correlations between RCC1/SNHG3/SNHG12 and each molecule of the immune checkpoint were analyzed using the “Pancancer Analysis” module of the Sangerbox tool. Spearman correlation test was used to calculate *p*-values and partial correlation values.

### Gene enrichment analysis

In order to explore the association between gene expression and each cellular pathway, using the “Expression” module of GSCA tool, we selected “Expression and Pathway activity” and “GSVA and Pathway activity” and analyzed the association between individual RCC1/SNHG3/SNHG12 genes and pathway activity, respectively. After considering them as a gene set, their correlation with pathway activity was also analyzed.

For the BioGRID database (https://thebiogrid.org/) containing proteins that have been validated to interact with RCC1/SNHG3/SNHG12 in studies, the venn mapping tool in Hiplot online tool (https://hiplot.com.cn/basic/venn) was used cross-tabulations were performed and venn diagrams were generated. In addition, all proteins directly related to RCC1 from textmining, experiments, and databases were obtained from the STRING database (https://cn.string-db.org/). The proteins directly associated with SNHG3/SNHG12 interactions in RNAInter tool (http://www.rnainter.org/), EuRBPDB tool (http://eurbpdb.syshospital.org/) were pooled. And the proteins in the ceRNA network associated with SNHG3/SNHG12 were summarized in the ENCORI tool (https://starbase.sysu.edu.cn/). Similarly, the above results were cross-tabulated using the venn mapping tool and 24 key proteins were obtained. These 24 proteins were analyzed for Gene Ontology (GO) and KEGG pathway enrichment using the Sangerbox tool based on the R software clusterProfiler package, and bubble plots were generated. In addition, further validation was performed using David’s online tool (https://david.ncifcrf.gov/). After entering information on 24 proteins as well as RCC1/SNHG3/SNHG12, we also performed GO and KEGG analysis.

### Drug-related analysis

We tried to discover drug molecules that inhibit the expression of RCC1/SNHG3/SNHG12. Using the XenaShiny tool on the Hiplot website (https://hiplot.com.cn/advance/ucsc-xena-shiny), after selecting “CCLE: Drug Response Association” and “CCLE: Drug Response Difference” from the “Quick PanCan Analysis” module, differences and associations with drug-target responses in pan-cancer were explored by regarding RCC1/SNHG3/SNHG12 as a signature.

In addition, we used the “Drug” module of the GSCA tool to explore the correlation between RCC1/SNHG3/SNHG12 gene expression and Genomics of Therapeutics Response Portal (CTRP) drugs in pan-cancer by selecting the “CTRP drug sensitivity and expression correlation”, and listed the top 30 drugs.

## Data Availability

The datasets presented in this study can be found in online repositories. The names of the repository/repositories and accession number(s) can be found in the article/Supplementary Material.

## References

[B1] Avis J. M. Clarke P. R. (1996). Ran, a GTPase involved in nuclear processes: its regulators and effectors. J. Cell Sci. 109, 2423–2427. 10.1242/jcs.109.10.2423 8923203

[B2] Bai Z. Gust R. (2009). Breast cancer, estrogen receptor and ligands. Arch. Pharm. 342 (3), 133–149. 10.1002/ardp.200800174 19274700

[B3] Bischoff F. R. Ponstingl H. (1995). Catalysis of guanine nucleotide exchange of Ran by RCC1 and stimulation of hydrolysis of Ran-bound GTP by Ran-GAP1. Methods Enzymol. 257, 135–144. 10.1016/s0076-6879(95)57019-5 8583915

[B4] Boddicker N. J. Hu C. Weitzel J. N. Kraft P. Nathanson K. L. Goldgar D. E. (2021). Risk of late-onset breast cancer in genetically predisposed women. J. Clin. Oncol. 39 (31), 3430–3440. 10.1200/jco.21.00531 34292776 PMC8547938

[B5] Boudhraa Z. Carmona E. Provencher D. Mes-Masson A. M. (2020). Ran GTPase: A key player in tumor progression and metastasis. Front. Cell Dev. Biol. 8, 345. 10.3389/fcell.2020.00345 32528950 PMC7264121

[B6] Bray F. Laversanne M. Weiderpass E. Soerjomataram I. (2021). The ever-increasing importance of cancer as a leading cause of premature death worldwide. Cancer 127 (16), 3029–3030. 10.1002/cncr.33587 34086348

[B7] Cekan P. Hasegawa K. Pan Y. Tubman E. Odde D. Chen J. Q. (2016). RCC1-dependent activation of Ran accelerates cell cycle and DNA repair, inhibiting DNA damage-induced cell senescence. Mol. Biol. Cell 27 (8), 1346–1357. 10.1091/mbc.E16-01-0025 26864624 PMC4831887

[B8] Chen J. Wu Z. Zhang Y. (2019). LncRNA SNHG3 promotes cell growth by sponging miR-196a-5p and indicates the poor survival in osteosarcoma. Int. J. Immunopathol. Pharmacol. 33, 2058738418820743. 10.1177/2058738418820743 30791797 PMC6329016

[B9] Chen Q. Zhou W. Du S. Q. Gong D. X. Li J. Bi J. B. (2019). Overexpression of SNHG12 regulates the viability and invasion of renal cell carcinoma cells through modulation of HIF1α. Cancer Cell Int. 19, 128. 10.1186/s12935-019-0782-5 31114448 PMC6518781

[B10] Collina F. Aquino G. Brogna M. Cipolletta S. Buonfanti G. De Laurentiis M. (2019). LncRNA HOTAIR up-regulation is strongly related with lymph nodes metastasis and LAR subtype of Triple Negative Breast Cancer. J. Cancer 10 (9), 2018–2024. 10.7150/jca.29670 31205562 PMC6548158

[B11] Crotty S. (2011). Follicular helper CD4 T cells (TFH). Annu. Rev. Immunol. 29, 621–663. 10.1146/annurev-immunol-031210-101400 21314428

[B12] Dacheng W. Songhe L. Weidong J. Shutao Z. Jingjing L. Jiaming Z. (2020). LncRNA SNHG3 promotes the growth and metastasis of colorectal cancer by regulating miR-539/RUNX2 axis. Biomed. Pharmacother. 125, 110039. 10.1016/j.biopha.2020.110039 32187965

[B13] Dai G. Huang C. Yang J. Jin L. Fu K. Yuan F. (2020). LncRNA SNHG3 promotes bladder cancer proliferation and metastasis through miR-515-5p/GINS2 axis. J. Cell. Mol. Med. 24 (16), 9231–9243. 10.1111/jcmm.15564 32596993 PMC7417716

[B14] Dasso M. Nishitani H. Kornbluth S. Nishimoto T. Newport J. W. (1992). RCC1, a regulator of mitosis, is essential for DNA replication. Mol. Cell. Biol. 12 (8), 3337–3345. 10.1128/mcb.12.8.3337 1630449 PMC364581

[B15] Deng Y. Yu L. Zhao Y. Peng J. Xu Y. Qin J. (2021). RCC1 expression as a prognostic marker in colorectal liver Oligometastases. Pathol. Oncol. Res. 27, 1610077. 10.3389/pore.2021.1610077 34924821 PMC8674189

[B16] Eyster K. M. (2016). The estrogen receptors: An overview from different perspectives. Methods Mol. Biol. 1366, 1–10. 10.1007/978-1-4939-3127-9_1 26585122

[B17] Hegde S. Leader A. M. Merad M. (2021). MDSC: Markers, development, states, and unaddressed complexity. Immunity 54 (5), 875–884. 10.1016/j.immuni.2021.04.004 33979585 PMC8709560

[B18] Heinlein C. A. Chang C. (2002). Androgen receptor (AR) coregulators: an overview. Endocr. Rev. 23 (2), 175–200. 10.1210/edrv.23.2.0460 11943742

[B19] Hirahara K. Nakayama T. (2016). CD4+ T-cell subsets in inflammatory diseases: beyond the Th1/Th2 paradigm. Int. Immunol. 28 (4), 163–171. 10.1093/intimm/dxw006 26874355 PMC4889886

[B20] Hou J. Lin L. Zhou W. Wang Z. Ding G. Dong Q. (2011). Identification of miRNomes in human liver and hepatocellular carcinoma reveals miR-199a/b-3p as therapeutic target for hepatocellular carcinoma. Cancer Cell 19 (2), 232–243. 10.1016/j.ccr.2011.01.001 21316602

[B21] Hsu I. Yeh C. R. Slavin S. Miyamoto H. Netto G. J. Tsai Y. C. (2014). Estrogen receptor alpha prevents bladder cancer via INPP4B inhibited akt pathway *in vitro* and *in vivo* . Oncotarget 5 (17), 7917–7935. 10.18632/oncotarget.1421 25277204 PMC4202170

[B22] Huang W. Tian Y. Dong S. Cha Y. Li J. Guo X. (2017). The long non-coding RNA SNHG3 functions as a competing endogenous RNA to promote malignant development of colorectal cancer. Oncol. Rep. 38 (3), 1402–1410. 10.3892/or.2017.5837 28731158 PMC5549033

[B23] Jiang B. Hailong S. Yuan J. Zhao H. Xia W. Zha Z. (2018). Identification of oncogenic long noncoding RNA SNHG12 and DUXAP8 in human bladder cancer through a comprehensive profiling analysis. Biomed. Pharmacother. 108, 500–507. 10.1016/j.biopha.2018.09.025 30243082

[B24] Kumar S. Prajapati K. S. Singh A. K. Kushwaha P. P. Shuaib M. Gupta S. (2021). Long non-coding RNA regulating androgen receptor signaling in breast and prostate cancer. Cancer Lett. 504, 15–22. 10.1016/j.canlet.2020.11.039 33556545

[B25] Lan T. Ma W. Hong Z. Wu L. Chen X. Yuan Y. (2017). Long non-coding RNA small nucleolar RNA host gene 12 (SNHG12) promotes tumorigenesis and metastasis by targeting miR-199a/b-5p in hepatocellular carcinoma. J. Exp. Clin. Cancer Res. 36 (1), 11. 10.1186/s13046-016-0486-9 28073380 PMC5223416

[B26] Lee Y. S. Dutta A. (2009). MicroRNAs in cancer. Annu. Rev. Pathol. 4, 199–227. 10.1146/annurev.pathol.4.110807.092222 18817506 PMC2769253

[B27] Li D. Zhou W. Pang J. Tang Q. Zhong B. Shen C. (2019). A magic drug target: androgen receptor. Med. Res. Rev. 39 (5), 1485–1514. 10.1002/med.21558 30569509

[B28] Li Y. Zhao Z. Liu W. Li X. (2020). SNHG3 functions as miRNA sponge to promote breast cancer cells growth through the metabolic reprogramming. Appl. Biochem. Biotechnol. 191 (3), 1084–1099. 10.1007/s12010-020-03244-7 31956955 PMC7320061

[B29] Ma Q. Qi X. Lin X. Li L. Chen L. Hu W. (2020). LncRNA SNHG3 promotes cell proliferation and invasion through the miR-384/hepatoma-derived growth factor axis in breast cancer. Hum. Cell 33 (1), 232–242. 10.1007/s13577-019-00287-9 31586299

[B30] Moore J. D. (2001). The Ran-GTPase and cell-cycle control. Bioessays 23 (1), 77–85. 10.1002/1521-1878(200101)23:1<77:Aid-bies1010>3.0.Co;2-e 11135312

[B31] Pan M. Jiang C. Tse P. Achacoso N. Alexeeff S. Solorzano A. V. (2022). TP53 gain-of-function and non-gain-of-function mutations are differentially associated with sidedness-dependent prognosis in metastatic colorectal cancer. J. Clin. Oncol. 40 (2), 171–179. 10.1200/jco.21.02014 34843402 PMC8718185

[B32] Pilling A. Kim S. H. Hwang C. (2022). Androgen receptor negatively regulates mitotic checkpoint signaling to induce docetaxel resistance in castration-resistant prostate cancer. Prostate 82 (2), 182–192. 10.1002/pros.24257 34672379 PMC9298324

[B33] Read K. A. Powell M. D. Sreekumar B. K. Oestreich K. J. (2019). *In vitro* differentiation of effector CD4(+) T helper cell subsets. Methods Mol. Biol. 1960, 75–84. 10.1007/978-1-4939-9167-9_6 30798522

[B34] Ren X. Jiang K. Zhang F. (2020). The multifaceted roles of RCC1 in tumorigenesis. Front. Mol. Biosci. 7, 225. 10.3389/fmolb.2020.00225 33102517 PMC7522611

[B35] Riahi A. Radmanesh H. Schürmann P. Bogdanova N. Geffers R. Meddeb R. (2018). Exome sequencing and case-control analyses identify RCC1 as a candidate breast cancer susceptibility gene. Int. J. Cancer 142 (12), 2512–2517. 10.1002/ijc.31273 29363114

[B36] Richards K. E. Zeleniak A. E. Fishel M. L. Wu J. Littlepage L. E. Hill R. (2017). Cancer-associated fibroblast exosomes regulate survival and proliferation of pancreatic cancer cells. Oncogene 36 (13), 1770–1778. 10.1038/onc.2016.353 27669441 PMC5366272

[B37] Sahai E. Astsaturov I. Cukierman E. DeNardo D. G. Egeblad M. Evans R. M. (2020). A framework for advancing our understanding of cancer-associated fibroblasts. Nat. Rev. Cancer 20 (3), 174–186. 10.1038/s41568-019-0238-1 31980749 PMC7046529

[B38] Shan T. Chen S. Chen X. Lin W. R. Li W. Ma J. (2017). Prometastatic mechanisms of CAF-mediated EMT regulation in pancreatic cancer cells. Int. J. Oncol. 50 (1), 121–128. 10.3892/ijo.2016.3779 27878234

[B39] Siegel R. L. Miller K. D. Fuchs H. E. Jemal A. (2021). Cancer statistics, 2021. CA. Cancer J. Clin. 71 (1), 7–33. 10.3322/caac.21654 33433946

[B40] Song W. Craft J. (2019). T follicular helper cell heterogeneity: Time, space, and function. Immunol. Rev. 288 (1), 85–96. 10.1111/imr.12740 30874350 PMC6422039

[B41] Sung H. Ferlay J. Siegel R. L. Laversanne M. Soerjomataram I. Jemal A. (2021). Global cancer statistics 2020: GLOBOCAN estimates of incidence and mortality worldwide for 36 cancers in 185 countries. Ca. Cancer J. Clin. 71 (3), 209–249. 10.3322/caac.21660 33538338

[B42] Tamang S. Acharya V. Roy D. Sharma R. Aryaa A. Sharma U. (2019). SNHG12: An LncRNA as a potential therapeutic target and biomarker for human cancer. Front. Oncol. 9, 901. 10.3389/fonc.2019.00901 31620362 PMC6759952

[B43] Tesi R. J. (2019). MDSC; the most important cell you have never heard of. Trends Pharmacol. Sci. 40 (1), 4–7. 10.1016/j.tips.2018.10.008 30527590

[B44] Ur Rahman M. S. Cao J. (2016). Estrogen receptors in gastric cancer: Advances and perspectives. World J. Gastroenterol. 22 (8), 2475–2482. 10.3748/wjg.v22.i8.2475 26937135 PMC4768193

[B45] Vaquero J. Aoudjehane L. Fouassier L. (2020). Cancer-associated fibroblasts in cholangiocarcinoma. Curr. Opin. Gastroenterol. 36 (2), 63–69. 10.1097/mog.0000000000000609 31934895

[B46] Vineis P. Wild C. P. (2014). Global cancer patterns: causes and prevention. Lancet 383 (9916), 549–557. 10.1016/s0140-6736(13)62224-2 24351322

[B47] Wan Q. Tang M. Sun S. L. Hu J. Sun Z. J. Fang Y. T. (2021). SNHG3 promotes migration, invasion, and epithelial-mesenchymal transition of breast cancer cells through the miR-186-5p/ZEB1 axis. Am. J. Transl. Res. 13 (2), 585–600. 33594311 PMC7868844

[B48] Wang J. Z. Xu C. L. Wu H. Shen S. J. (2017). LncRNA SNHG12 promotes cell growth and inhibits cell apoptosis in colorectal cancer cells. Braz J. Med. Biol. Res. 50 (3), e6079. 10.1590/1414-431x20176079 28225893 PMC5333723

[B49] Wang O. Yang F. Liu Y. Lv L. Ma R. Chen C. (2017). C-MYC-induced upregulation of lncRNA SNHG12 regulates cell proliferation, apoptosis and migration in triple-negative breast cancer. Am. J. Transl. Res. 9 (2), 533–545. 28337281 PMC5340688

[B50] Wu J. Liu L. Jin H. Li Q. Wang S. Peng B. (2019). LncSNHG3/miR-139-5p/BMI1 axis regulates proliferation, migration, and invasion in hepatocellular carcinoma. Onco. Targets. Ther. 12, 6623–6638. 10.2147/ott.S196630 31692508 PMC6708045

[B51] Xu B. Mei J. Ji W. Bian Z. Jiao J. Sun J. (2020). LncRNA SNHG3, a potential oncogene in human cancers. Cancer Cell Int. 20 (1), 536. 10.1186/s12935-020-01608-x 33292213 PMC7640707

[B52] Yip C. H. Rhodes A. (2014). Estrogen and progesterone receptors in breast cancer. Future Oncol. 10 (14), 2293–2301. 10.2217/fon.14.110 25471040

[B53] Yudin D. Fainzilber M. (2009). Ran on tracks--cytoplasmic roles for a nuclear regulator. J. Cell Sci. 122, 587–593. 10.1242/jcs.015289 19225125

[B54] Zeng X. Zhong M. Yang Y. Wang Z. Zhu Y. (2021). Down-regulation of RCC1 sensitizes immunotherapy by up-regulating PD-L1 via p27(kip1)/CDK4 axis in non-small cell lung cancer. J. Cell. Mol. Med. 25 (8), 4136–4147. 10.1111/jcmm.16383 33630417 PMC8051708

[B55] Zhang C. Qu Y. Xiao H. Xiao W. Liu J. Gao Y. (2019). LncRNA SNHG3 promotes clear cell renal cell carcinoma proliferation and migration by upregulating TOP2A. Exp. Cell Res. 384 (1), 111595. 10.1016/j.yexcr.2019.111595 31505165

[B56] Zhang P. F. Wang F. Wu J. Wu Y. Huang W. Liu D. (2019). LncRNA SNHG3 induces EMT and sorafenib resistance by modulating the miR-128/CD151 pathway in hepatocellular carcinoma. J. Cell. Physiol. 234 (3), 2788–2794. 10.1002/jcp.27095 30132868

[B57] Zhang C. Ren X. Zhang W. He L. Qi L. Chen R. (2020). Prognostic and clinical significance of long non-coding RNA SNHG12 expression in various cancers. Bioengineered 11 (1), 1112–1123. 10.1080/21655979.2020.1831361 33124951 PMC8291808

[B58] Zhao Q. Wu C. Wang J. Li X. Fan Y. Gao S. (2019). LncRNA SNHG3 promotes hepatocellular tumorigenesis by targeting miR-326. Tohoku J. Exp. Med. 249 (1), 43–56. 10.1620/tjem.249.43 31548493

[B59] Zheng S. Jiang F. Ge D. Tang J. Chen H. Yang J. (2019). LncRNA SNHG3/miRNA-151a-3p/RAB22A axis regulates invasion and migration of osteosarcoma. Biomed. Pharmacother. 112, 108695. 10.1016/j.biopha.2019.108695 30797154

[B60] Zhou J. Teng R. Xu C. Wang Q. Guo J. Xu C. (2013). Overexpression of ERα inhibits proliferation and invasion of MKN28 gastric cancer cells by suppressing β-catenin. Oncol. Rep. 30 (4), 1622–1630. 10.3892/or.2013.2610 23843035

[B61] Zhou B. Li L. Li Y. Sun H. Zeng C. (2018). Long noncoding RNA SNHG12 mediates doxorubicin resistance of osteosarcoma via miR-320a/MCL1 axis. Biomed. Pharmacother. 106, 850–857. 10.1016/j.biopha.2018.07.003 30119255

[B62] Zhou S. Yu L. Xiong M. Dai G. (2018). LncRNA SNHG12 promotes tumorigenesis and metastasis in osteosarcoma by upregulating Notch2 by sponging miR-195-5p. Biochem. Biophys. Res. Commun. 495 (2), 1822–1832. 10.1016/j.bbrc.2017.12.047 29229388

