# Supplementary material for: Systematic pan-cancer analysis on the expression and role of regulator of chromatin condensation 1/small nucleolar RNA host gene 3/small nucleolar RNA host gene 12
Source: Front Mol Biosci. 2022 Sep 6;9:946507. doi: 10.3389/fmolb.2022.946507 (PMC9486007; doi:10.3389/fmolb.2022.946507)
Supplement: Supplementary file 1 [file Image1.pdf]

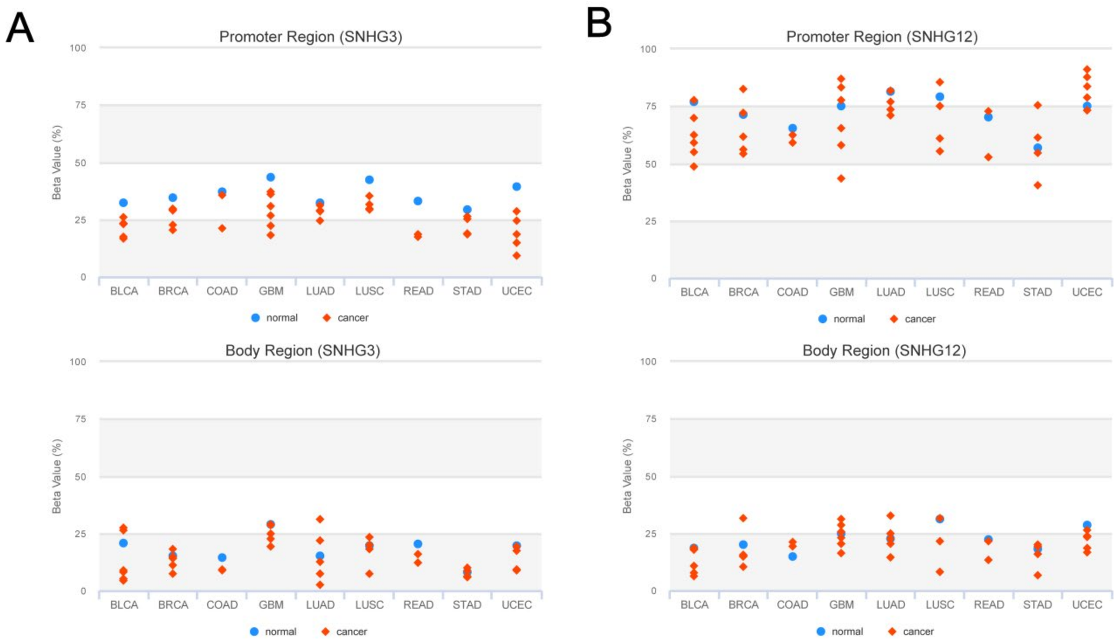

Supplementary Figure1. Methylation of SNHG3 and SNHG12 in cancers. (A) SNHG3. (B) SNHG12.

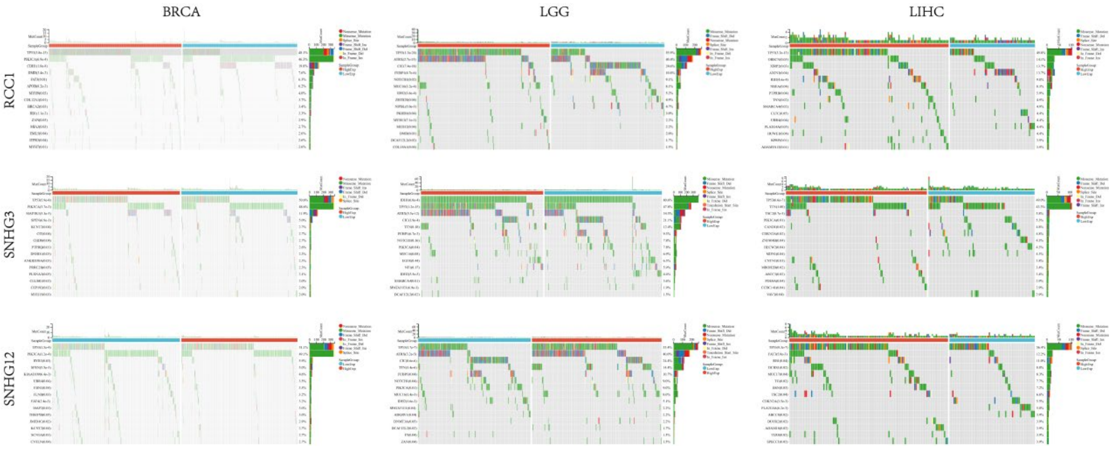

Supplementary Figure2. Tumor mutation landscape of RCC1/SNHG3/SNHG12 EXP in BRCA/LGG/LIHC.

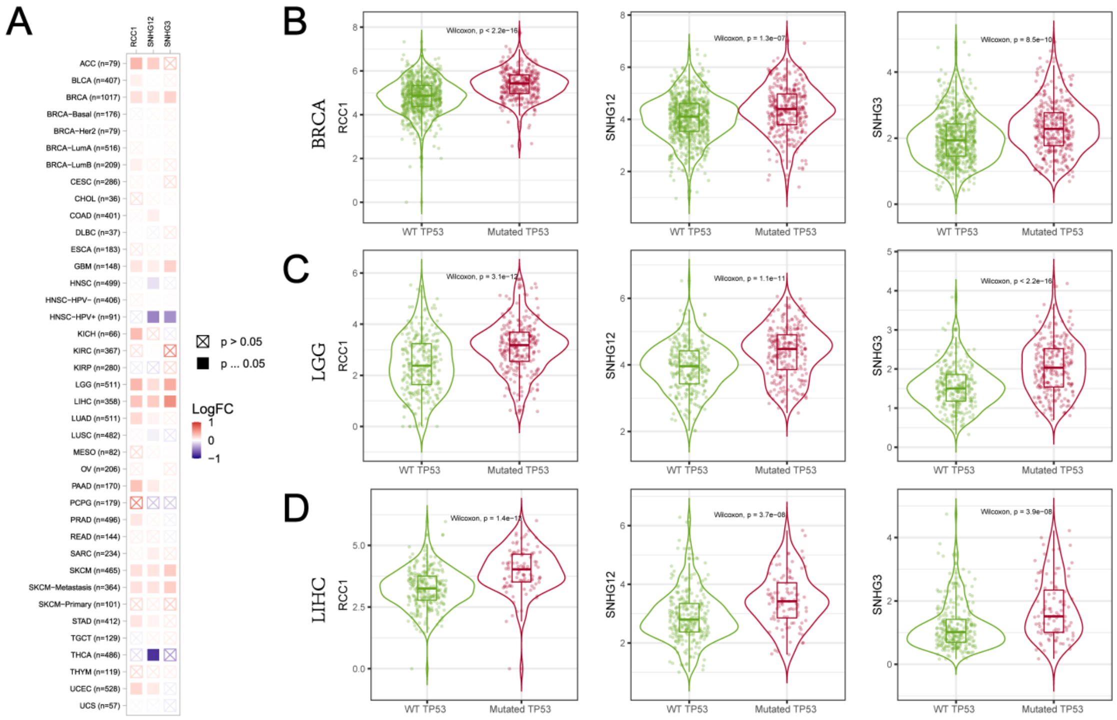

Supplementary Figure3. Correlation between RCC1/SNHG3/SNHG12 and TP53 in cancers. (A) Correlation of RCC1/SNHG3/SNHG12 and TP53 expression in different cancers. (B-D) Differential expression of RCC1/SNHG3/SNHG12 in WT TP53 and Muted TP52 in BRCA, LGG and LIHC.

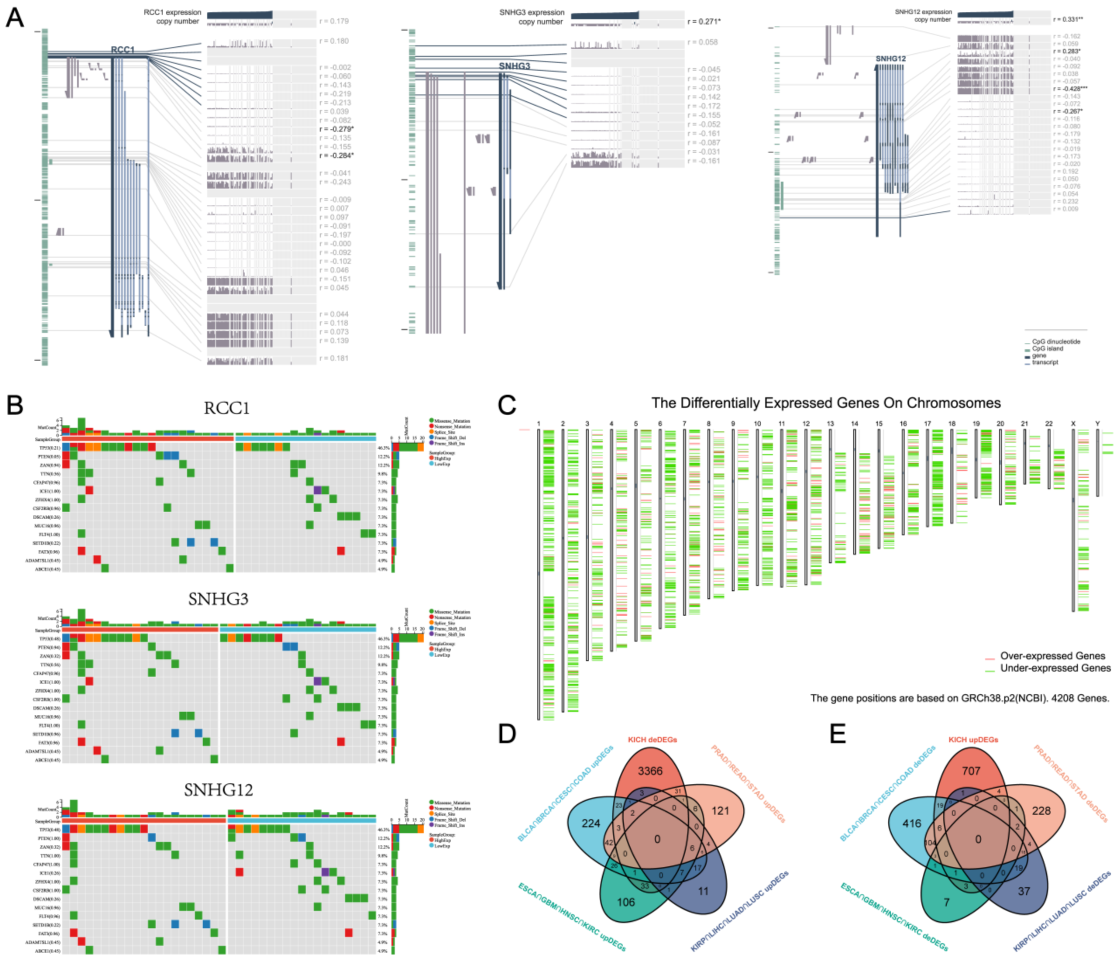

Supplementary Figure4. Analysis of RCC1/SNHG3/SNHG12 in KICH. (A) Methylation of RCC1/SNHG3/SNHG12 in KICH. (B) Tumor mutation landscape of RCC1/SNHG3/SNHG12 EXP in KICH. (C) Overall differential genes in KICH tumors with their chromosomal distribution. (D,E) Venn diagram of KICH and other cancer differential genes.

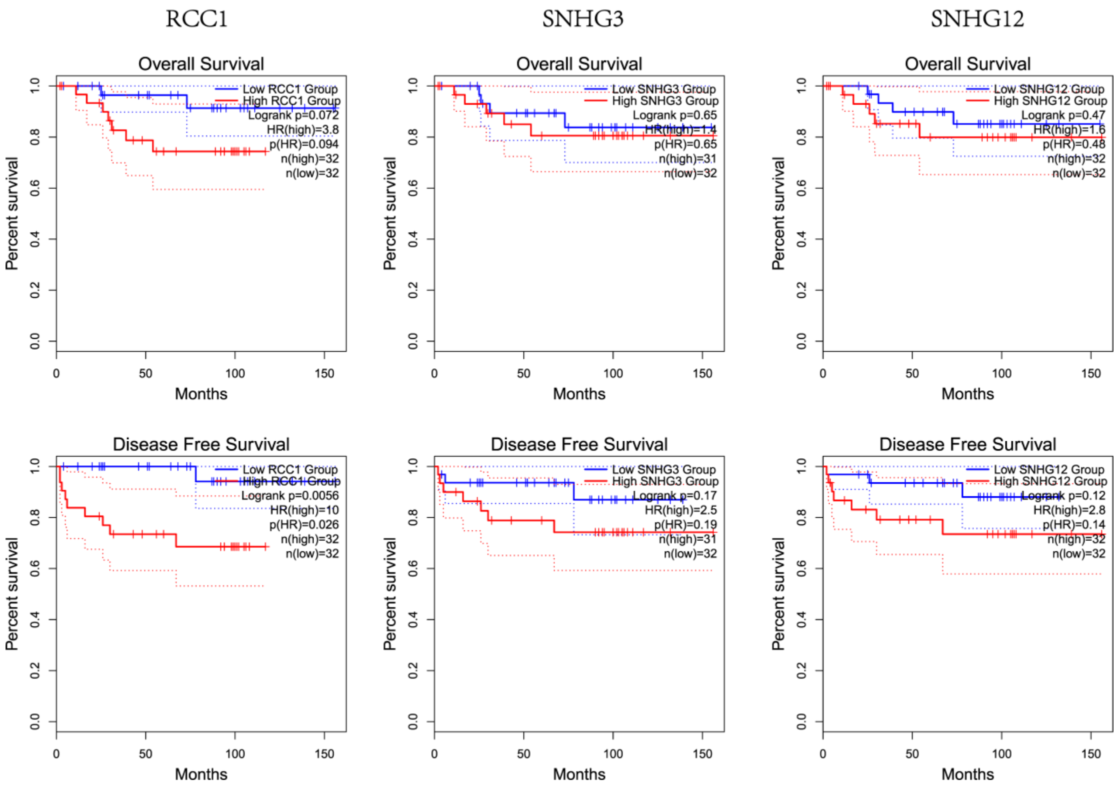

Supplementary Figure5. OS and DFS of RCC1/SNHG3/SNHG12 in KICH.

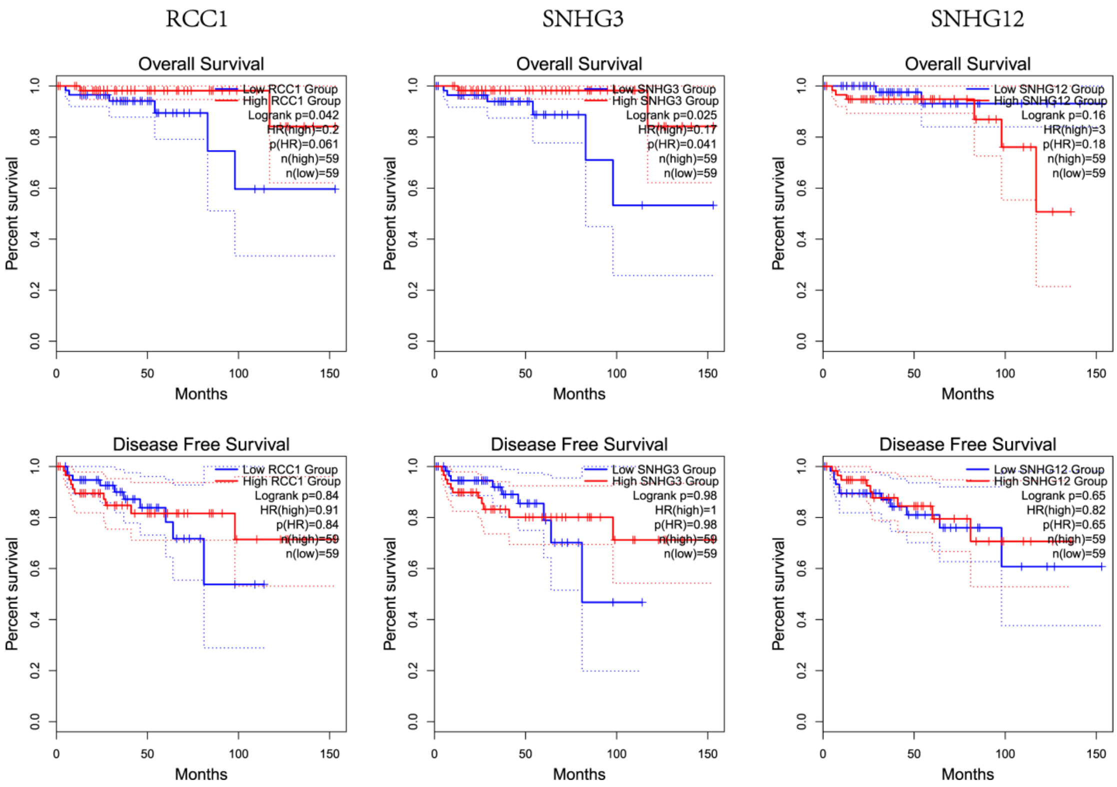

Supplementary Figure6. OS and DFS of RCC1/SNHG3/SNHG12 in THYM.

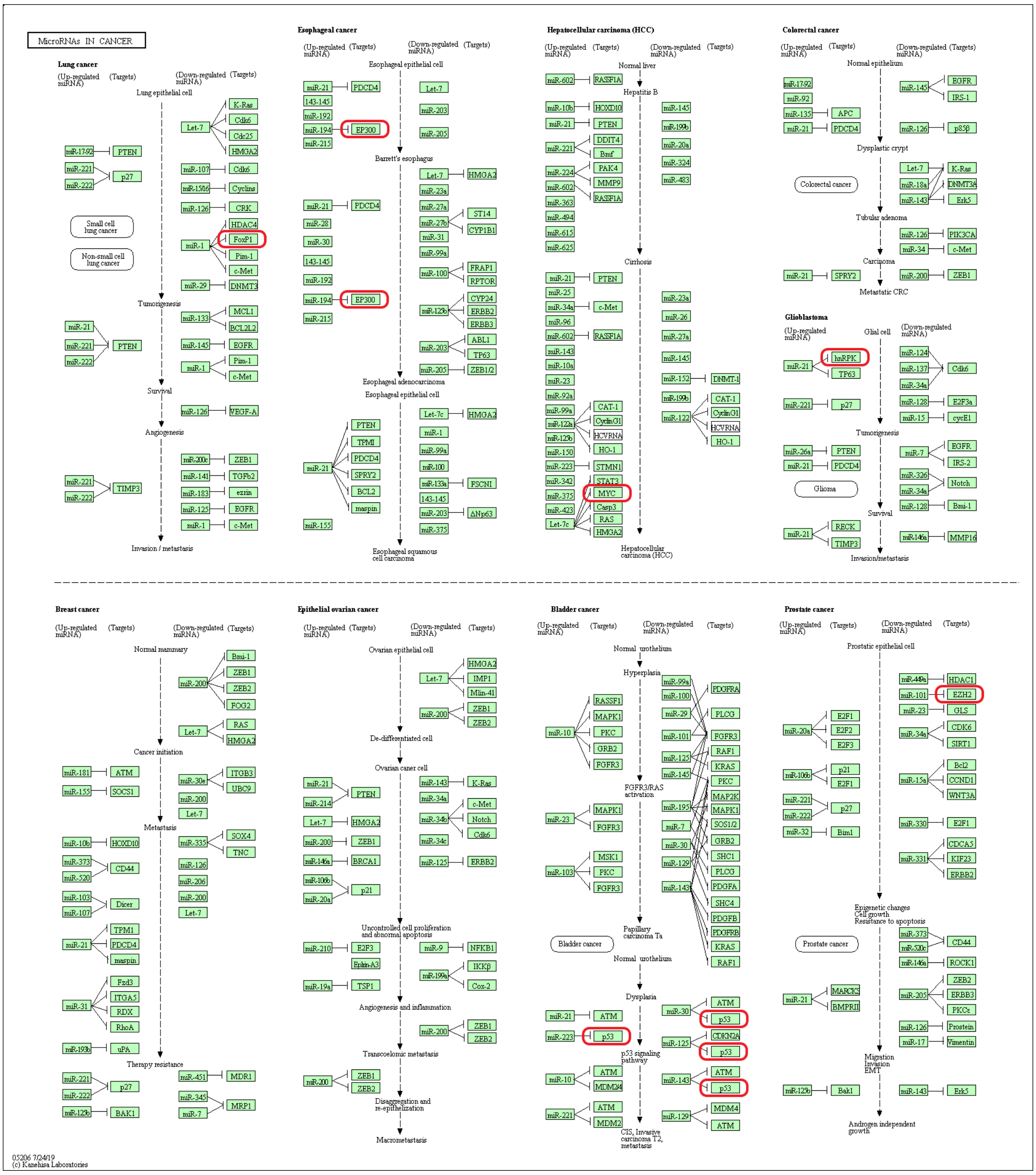

Supplementary Figure7. Proteins associated with RCC1/SNHG3/SNHG12 are involved in the MicroRNAs in cancer pathway.
